
JOURNAL INFORMATION
==============================
NLM Title Abbreviation: Med Chir Rev
NLM Journal ID: 0034617

The Medico-Chirurgical Review

Publisher: Biomedical Journal Digitization Project

ARTICLE INFORMATION
==============================
PMCID: PMC4966176
PMID: 29917599
Article version: 1
Subjects: Reviews

Drs. Blumenbach and Elliotson on Physiology

Print publication date: 1828 Jul 1
Volume: 9
Issue: 17
First page: 97
Last page: 120
1828 Jun 7
License: This work is free of known copyright restrictions. For more information, please see PMC Back Issue Digitization.
License URL: https://creativecommons.org/publicdomain/mark/1.0/

==============================

### XI.

*Elements of Physiology.* By J. FRED. BLUMENBACH, M.D. F.R.S. Professor of Medicine in the University of Göttingen; translated from the Latin of the Fourth and Last Edition, by JOHN ELLIOTSON, M.D. Cantab. Fellow of the Royal College of Physicians; Physician to, and Lecturer on, the Practice of Medicine in St. Thomas's Hospital.

———Latas a Numine leges
Religiosa doce, mentesque cupidine veri
Allice.

LORD BACON tells us that some books may be tasted, some may be chewed, and some few may be swallowed and digested. They, indeed, on which the latter process can be performed are exceedingly few; but when met with, the person who has enjoyed the feast, should, in the goodness of his heart, present the same to his fellow creatures, in order that they, with him, should have the pleasure of drawing thence their nutriment. This is exactly our feeling in presenting to our readers the work mentioned above; a work, the merits of which are of the highest order. Blumenbach is its author; a man, known as being possessed of all the preliminary knowledge necessary to the formation of a good physiologist. Comparative anatomy is his delight; he is a good linguist; has read much; has a sound judgment; and seems to be a sincere lover of truth. Another peculiar advantage is, his freedom from any particular theory or hypothesis. He has, what may be called, a width of mind; his intellectual grasp is truly extensive; and he does not, like many of our medical physiologists, begin theorizing with a single fact for the base of a system. He has the ability to analyse; and the effect of this is, that he arranges well, and defines, where he attempts definition, (which is not often,) with the most consummate skill.

Such is Blumenbach. A work, a foreign work in particular, labours under many disadvantages. Every country has a different mode of thinking: and often the individuals of one do not perceive the beauties of the views of the individuals of another, simply because these views are not presented to them in the way in which they have been accustomed to receive impressions and ideas. Another disadvantage arises from the circumstance of the dependence of the work upon the merits of a translator. The meaning of an author is often entirely altered, simply by the ignorance of the person who engages to put the original into a different language; his views are perverted; his illustrations are deprived of all their force; and a prejudice is thus raised against the work itself, that impedes, should the original be a valuable production, the progress of knowledge, by keeping this product of a gigantic and well exercised mind from being contemplated in all the graces of its true character by the minds of others who can appreciate with accuracy and judge with fairness. Fortunately for science, Blumenbach's work has not been presented to the medical men of Britain under such circumstances. An individual, well known for his general attainments as a physician and as a scholar, has turned his attention to this work of translation; and, in tracing the thoughts of his author, has entered with the spirit of a true philosopher into the subject before him, and has shewn the attentive delight excited in his mind by the rich notes with which he has studded the original. This individual is Dr. Elliotson; whom we are delighted to see engaged in what, by some, may be thought the menial office of a translator, simply on the belief that, as a fool is the best judge of a fool, so a wise man of a wise man.

But now to the work itself: which is divided into 44 sections, each of which is interesting: some more so than others.

The first section is upon the living human body *generally*, in the consideration of which Blumenbach points out three objects as worthy of attention: first, the *materials* of its subsistence, afforded by the fluids; second, the *structure* of the solids, containing the fluids; and third, the *vital powers*. In a note on this section, we have given to us a brief and clear account of the different arrangement of *elementary tissues* of which the organs of the body are composed; also of the proximate and ultimate principles of animal bodies; as well as those of vegetables.

Being thus introduced, an account is given in the second section of the *fluids* of the body; the classification of which is excellent: namely, the *crude*, as the chyle, and matters absorbed on the surface and conveyed to the chyle: the *blood* itself: those *secreted* from the blood.

Our physiologist then turns his attention to the *blood* in particular, the importance, the physical and chemical characters of which are succinctly enumerated. A curious fact is stated respecting the vapour, which is emitted from blood while still warm, namely, that it affords a nidorous smell, which is most remarkable in the blood of carnivorous animals. In a note are stated the various opinions of physiologists respecting the quantity of blood in a well-formed adult; the lowest estimate being that of eight pounds, the highest 100.

Blumenbach maintains, that aeriform fluids in the *elastic state* cannot exist in the blood, since he found that a small portion of the purest air, infused into the jugular vein, excited palpitations, drowsiness, convulsions; and if the quantity was a little increased, death ensued. In a note by Dr. Elliotson, the facts stated make us conclude that the above opinion of Blumenbach must admit of limitation, since from Dr. Elliotson's statements we infer, that the injurious effects of the injection of air are dependent on the *rapidity* of the process. One fact cannot be passed unnoticed: it is this, that "if warm water is introduced, (an equal quantity of blood being first removed to prevent over-distention,) mere debility ensues, proportionate to the quantity: but if oils, or mucilages, or an inert impalpable powder are injected, life is at once destroyed by the obstruction of the minute ramifications of the pulmonary artery." Dr. Elliotson then gives a brief but excellent sketch of the different theories respecting the coagulation of the blood, to which we must refer our readers, and take this opportunity of pointing out one circumstance, leading the mind to admire the wisdom of the Creator. It is this, "Blood almost always coagulates also in the vessels running through healthy parts to others in a state of mortification, and in large vessels adjoining a pulmonary abscess ; in which cases, the final cause, prevention of hæmorrhage, is evident."

In a note on the blood of brutes in relation to the blood of man, the translator introduces all that is known respecting *transfusion*, noticing the experiments of Dr. Blundel, a gentleman who has saved many women by the process of transfusion from death, to the danger of which they have, by excessive uterine hæmorrhage, been exposed.

The next section presents to our view *the solids*, their chemical composition, their structure, &c. Blumenbach then describes the general diffusion, the peculiarities, the offices of the cellular tissue, which he calls the *mucous web;* and from the facts respecting this web, he concludes, "First, that this membrane is so fundamental a constituent of our structure, that, were every other part removed, and it to retain its position, the body would preserve its form ; secondly, that it forms a connexion and sort of passage between all parts of the system, however different from each other in nature, or remote in situation : a circumstance worthy of attention, as putting an end to the verbal disputes respecting the continuation of membranes, and affording an explanation of many morbid phenomena." The last remark is one worthy of a Blumenbach.

This physiologist maintains in addition, that this mucous web (Dr. E. prefers *cellular tissue*, because Blumenbach uses the term *vis cellulosa*, referring to a power possessed by this membrane) is derived from the lymph of the blood, "for I have seen lymph," says he, "transuded on the surface of inflamed lungs, and changed into this mucous web, which, by forming false membranes, unites these organs to the pleura."

The fourth section brings us to the *experimentum crucis* of every physiologist, VITALITY. Blumenbach has his opinions on this matter ; which may be stated to be the following, previously noticing his introductory observation on this point.

"Although vitality is one of those subjects which are more easily known than defined, and usually, indeed, rendered more obscure rather than illustrated by an attempt at definition, its effects are sufficiently manifest and ascribable to peculiar *powers* only. The epithet *vital* is given to these powers, because on them so much depend both the actions of the whole body during life, and those which remain in some parts for a short time after death, that they are not referable to any qualities merely physical, chemical, or mechanical."

Blumenbach's peculiar ideas are stated in the following passage :—

"As to the question so long agitated by physiologists—whether the diversity of the phenomena exhibited in the similar parts of the living solid is to be attributed to modifications only, or to distinct species of the vital energy, we think it best to establish *distinct orders of the vital powers*, according to the variety of phenomena by which they are manifested. These phenomena are threefold according to him :—organic *formation* and increase ;—*motion* in the parts when formed ;—*sensation* from the motion of certain similar parts."

The first gives the determinate *form* which every body has ; and this species of the vital powers, producing the genital and nutritive fluids, and preparing them for organic nature, Blumenbach calls the *nisus formativus*. But this nisus formativus cannot be considered as essentially a vital power, because we find a certain formative power exhibited in inorganic matter in the formation of crystals. This nisus formativus must therefore be considered as modified.

The vital powers manifested by *motion*, Blumenbach divides into *common*, as those belonging to parts widely distributed: such as contractility to the mucous web;—irritability to the muscular fibre;—and into *proper*;—those possessed by some particular organs only, giving to them, for the purpose of particular anomalous actions, as the motions of the iris—the erection of the nipple. This contractility of the mucous web, we have already mentioned, our physiologist names it the *vis cellulosa:* and the end of this, he supposes to be to propel the serous vapour into the lymphatic system.

As regarding *sensation*, the third vital power, called also *sensibility*, also *vis nervea*, "is the cause of perception in the mind, when irritation is excited in parts to which it is distributed."

These views regarding vitality have two recommendatory circumstances:—the first, that in this order we have the gradual succession followed in our formation: the second, that we have something tangible presented to our view.

Indeed, this last circumstance is one to which men at last are turning their attention. The human intellect is beginning to be freed from the trammels of superstition, which would, if possible, oblige men to think only in one way, and which delights to clothe palpable facts and sensible manifestations with a spiritual halo; as if truth could be more sacred when thus surrounded, than when clothed in her simple beauty. The discussions about the *essential nature* of life are, we trust, dying away. We now are learning to know life by its manifestations, and all that is important for us as practitioners to know is, that this life, whatever it be, is exhibited through parts, called *organs*, and that when these organs are in a sound state, then the vital powers are in harmony;—when the former are diseased or injured, the vital powers are affected, which affection must be removed, not by spiritual discourses on life, but by removing the diseased state of the organ. It would be a great blessing were physiologists, before entering upon this question of life, to ask themselves *quid utile?*

With respect to the vitality of the two great divisions of matter, Blumenbach seems to be of the opinion that *solids* have, as their peculiar property, vitality; and that those fluids, thought to have vitality, have so only from the solids they contain. This celebrated physiologist disputes the vitality of the blood; for his arguments on this head the reader must examine the work itself. Dr. Elliotson differs from him, and considers "that fluids as well as solids are susceptible of life;" and, at the end of this section, makes some interesting remarks on sympathy.

The fifth section embraces the mental faculties, a subject till of late not sufficiently attended to by the physiologist. These he arranges under two heads, the *intellectual faculty* and *appetency;* more correctly and simply stated, the *intellectual* and *affective* faculties. Under the former are ranged *perception*, *memory*, *attention*, *imagination*, *abstraction*, *judgment*, and *reason*; under the latter, *desires* and *aversions*. Blumenbach may be said to have a third faculty, namely, *volition*.

This brief view given by Blumenbach is quite insufficient to explain the varied phenomena of the mind presented in its healthy and diseased exhibitions; and Dr. Elliotson has, we are exceedingly glad to see, supplied the want by a clear and full account of Gall's *theory* (not *hypothesis*); in which is shadowed out the best system of the human mind. Every one indeed should read the terse and excellent note at the end of this section. It will amply repay the perusal. We could extract it with pleasure. But we hope that this review will have the effect of an appetizer, to lead every one to enjoy the book for himself. But if this note may be with propriety recommended to notice, much more may those which are attached to the next section on health and human nature. We see in these the philosopher; a man, who ventures to stand forward in defence of his opinions, and does not shrink back for fear of consequences: a man, who goes to the tribunal of facts and hears the judgment of his own mind thereon, and obeys. And although we may not go in with every expression or opinion that Dr. Elliotson states in these notes, we testify, that as a whole, we consider the same as a master-piece, and will stamp the writer as an individual, who *can* think, *will* think, and *does* think. We detest that doctrine, too prevalent even now, that it is better to labour under delusion, than to advocate and hold forth truth, because these certain truths may be dangerous. Ignorance we have always considered as an evil; —even, in some cases, as a crime. So we trust, ever to consider it, and any individual, whoever he be, that endeavours to light the torch of truth at the funeral pile of error, we shall hail with respect and with joy.

Blumenbach states four things as necessary to health:—fluids properly prepared—solids duly formed from the fluids—the invigorating influence of the *vital powers* upon the solids—lastly, a sound mind in a sound body. Considering these necessary circumstances, Galen might well assert that no one enjoys perfect health. Health is variously modified: the modifications are the *temperaments*, which are four—the *sanguineous*, the *choleric*, the *melancholic*, and the *phlegmatic*, each of which has its peculiar characteristics. This doctrine of the temperaments, though quite distinct, has been associated with *humoral pathology;* and medical men, in neglecting the one, have disregarded the other. Hence the little attention paid to temperaments, a want of attention which is the source of numberless evils.

Under this section, Blumenbach notices the general divisions of functions, into the *vital*, the *animal*, the *natural*, and the *genital*, which have nothing to recommend them except in aiding the memory, as these divisions are not according to nature. In the note A to this section, Dr. Elliotson gives a new view of temperaments, devised by Dr. Thomas, of Paris, who arranges the temperaments according to the predominance of the head, chest, or abdomen. p. 54. We again recommend to the perusal of every one the notes of this section.

In the next section Blumenbach examines, with the candour of a sincere observer of nature, the blood in *its motion:* and divides the subject, quite logically, into two parts; first, the *vessels* containing the blood; second, the *powers* propelling it. In speaking upon the arteries, this celebrated physiologist disputes the commonly-received notion, that the united capacity of the *branches*, in any part of the sanguiferous system, is greater than that of the trunk whence they arise. Blumenbach adds—

"But I fear that this is too general an assertion, and even that the measure of the diameter has been sometimes improperly confounded with that of the area. I myself have never been able to verify it, although my experiments have been frequently repeated, and made, not in vessels injected with wax, after the bad example of some illustrious physiologists, but on the undisturbed vessels of recent subjects, *v. c.* on the innominata and its two branches, the right carotid and subclavian—on the brachial and its two branches, the radial and ulnar."

Blumenbach further remarks respecting this matter:—

"The inconstancy of the proportion between the capacity of the branches and that of the trunks, is clearly shewn by the various sizes of the vessels under different circumstances, *v. c.* by the relative capacity of the inferior thyroid artery in the infant and the adult; of the epigastric artery in the virgin and the mother near her delivery; and also of the uterine vessels in the vagina and the pregnant woman; of the omental vessels during the repletion and vacuity of the stomach."

Blumenbach maintains that, in the systole of the ventricles, the external portions of the heart are drawn towards their septum, and the apex of the heart towards the base.

"This, at first sight," adds this physiologist, "seems disproved, by the circumstance of the apex striking against the left nipple, and, consequently, appearing elongated,—a circumstance, however, attributable to the double impetus of the blood flowing into the auricles and expelled from the ventricles, by which double impetus the heart must be driven against that part of the ribs."

Besides the heart as the great moving power, other *secondary* powers exist: such are the *arteries*, having a muscular coat, being irritable, and being so well supplied with nerves in the lower part of the abdomen: the *veins* are added to these, since, from the circumstances of these performing vital functions in the liver and placenta, we must be convinced that they have vital powers. Besides, there is a muscular layer in the extreme veins near the heart. Blumenbach, it would appear, is of the opinion, that the progression of the blood in the arteries is a kind of *oscillation*. What is called the *venous pulse*, Blumenbach states to be corresponding to respiration, and not to the action of the heart.

In the notes to this section, by Dr. Elliotson, we have presented to us a brief, but full, outline, of all that has been said upon the actions of the heart, and upon the irritability of arteries; also upon the structure of the hearts of different animals. The note K we must introduce.

"It would not be right to terminate this section without a note upon the discovery of the circulation of the blood; a truth of which the ancients are thought to have remained ignorant, from finding the arteries empty after death, but it was known that these contained blood during life. The discovery was made by our countryman, Dr. Harvey, Physician to St. Bartholomew's Hospital, and promulgated by him, at the age of forty-one, in an anatomical and surgical course of lectures at the College of Physicians, in 1619. He is entitled to the glory of having made it, says Hume, 'by reasoning alone, without any mixture of accident.' He informed Bayle, that he was led to it by reflecting on the arrangement of the valves of the heart and veins, as exhibited by his master Fabricius. Nothing, he knew, was planned in vain, and they clearly allowed a fluid to pass but one way. By this argument, and the fact of a ligature upon an *artery* causing the blood to accumulate in it on the side *nearest* the heart, and, upon a *vein*, *beyond* the ligature; and that animals bleed to death by wounds in arteries or veins, he chiefly established his doctrine. After his time, it was demonstrated with the microscope in cold-blooded animals. His immediate reward was general ridicule and abuse, and a *great* diminution of his practice; and no physician in Europe, who, at the time, had reached forty years of age, ever, to the end of life, adopted his doctrine of the circulation of the blood. (Hume's History, chap. 62.) When the truth could be denied no longer, he was pronounced a plagiarist; the circulation was declared to have been known to Plato, nay, more, to King Solomon. The circulation through the lungs had certainly been imagined by Servetus, a Spanish physician, who was slowly burnt to death by Calvin, for not being of the same opinion as himself upon a point in divinity."

This interesting statement should be a warning to those who oppose every thing that is new: and who, because a matter was not found out before, very coolly condemn it. It also is a verification of the well-known observation of Locke:—"Truth scarce ever yet carried it by vote any where, at its first appearance. New opinions are always suspected, and usually opposed, without any other reason, than because they are not common. But truth, like gold, is not the less so for being newly brought out of the mine. 'Tis trial and examination must give it price, and not any antique fashion: and though it be not yet current by the public stamp, yet it may, for all that, be as old as nature, and certainly not the less genuine."

With respect to animal heat, which forms the subject matter of the tenth section, we are informed that Haller maintained, that man cannot live in a temperature exceeding his own: an assertion now completely disproved. Man, indeed, can live in every climate: we see him in the Torrid and in the Frigid Zone. Blumenbach's explanation of the equable temperature of the body in all countries, is somewhat similar to that of Dr. Crawford's, namely, that oxygen becomes solidified, is carried through the system, acquires carbon in the small vessels, and thus sets free much of the latent heat which it had received. Besides this cause, our physiologist mentions two others, namely, the *corium* covering the body, and the *alimentary canal*, both of which, abundantly supplied with blood-vessels, seem to be almost as subsidiary lungs; and mentions cases where the lungs have been almost totally consumed, and yet the patients have existed: remember, says he, the coldness of the dog's nose; the burning, at one time of the cheeks, and, at another, of the palms of the hands, in hectic fever.

In the 12th section, Blumenbach proceeds to the consideration of the functions, by which mind and body are connected, and hence called *animal;* the organs of these being the brain, the spinal marrow, together with the nerves; the functions themselves being divisible into two principal classes, *sensorial* and *nervous;* "the former comprehending all excepting the nerves and their immediate origin; all that serves more directly as the connexion between the office of the nerves and the faculties of the mind."

Blumenbach notices the motion of the brain, which he maintains, is correspondent to respiration: and states a fact strikingly illustrative. p. 192.

As a proof of the manly intrepidity of Blumenbach, we may quote the following passage; "the spinal marrow is continuous with the brain, and may be said either to spring from the brain, as from a root, or, on the contrary, to *terminate in it*, and grow into its substance." And in support of the latter opinion, Gall is quoted. This seems to us manly: and it will gain for its author fame, when many physiologists, infinitely inferior to the one before us, who have refused to do justice to Gall for his discoveries, will be remembered, if at all, as the creatures of prejudice and the enemies of truth.

Speaking of the dissections of living animals, with the view of discovering the nerves, our physiologist makes some remarks which Magendie, Fleurens, Orlando, and other immolators at the altar of experiment (this seems scarcely a right name) would do well to remember.

"I am every day more convinced, that much caution and practice, and repetition of the same experiments in many different kinds of animals, are necessary in establishing the laws of physiology from dissections of living animals. To adduce the example of the supposed feeling of the medulla, I have found different results in many mammalia and birds. Many allowed the medulla to be destroyed without evincing any symptom of pain: others were convulsed, and cried out on the approach of the instrument. The latter might be agitated from the dread of fresh torment, on seeing the knife; and the former, having suffered great torture, might have been insensible to the less violent irritation of the medulla, even though it be endowed with nerves."

At the 221st page we have the following note, which we think conclusive upon this mode of experimenting.

"Fontana says, that after removing the brain of a turtle, and entirely emptying the cranium, the animal lived six months, and walked as before. M. Rolando attempted the experiment repeatedly, but the animal always died as soon as a cut was made behind the cerebellum. M. Rolando, says, 'he made innumerable experiments upon goats, lambs, pigs, deer, dogs, cats, and guineapigs, to ascertain the results of lesion of the tubercles, and parts near the optic thalami, but *rarely obtained the same results*.' M. Rolando, says, that lesion of the thalami optici, causes convulsions, M. Fleurens denies it (Gall, l. c. vi. p. 191.) M. Rolando found an unsteadiness, like that of intoxication, follow the removal of two-thirds of the lobes of the cerebrum in a chicken. M. Fleurens declares he must have wounded the cerebellum. M. Fleurens protests that the experiments of M. Rolando are contradictory to each other. (p. 215.) And after finding a chicken walk, fly, and swallow, shake its wings, and clean them with its beak, subsequently to losing the hemispheres of its brain, infers, that these are the residence of the understanding and feelings: the cerebellum, according to him, is destined to balance, to regulate motion; yet birds, after losing these parts, pecked and clawed their enemies, and perched.' (p. 266.) M. Rolando considers muscular action to depend upon the cerebellum, yet Magendie found animals perform regular motions after losing this part."

Speaking of the origin of the nerves, Blumenbach states, "some nerves, however, may be more properly considered as *uniting* with the brain and spinal marrow, than springing from them." This observation we have noticed with the view of endeavouring to give to every individual his due. Gall was the first who shewed that the nerves and spinal marrow only *communicate* with the brain; and this doctrine, though taught by him with the utmost clearness, is so far unknown, that a lecturer on anatomy has of late, so it has been stated to us, been claiming to himself the important discovery, that the sympathetic does not arise from the two twigs said to give origin to it, but that these are merely *communicating* branches. It is true, as Dr. Armstrong has observed, that two astronomers (unknown to each other,) in different countries, may see the same stars; so it may be with this anatomist and Gall: but the priority of observation surely belongs to the latter individual. Indeed, what is more, Bichat makes an evident distinction between the *cerebral* and *ganglionic* system. The former, according to him, presiding over ANIMAL life; the latter over ORGANIC; because this latter system belongs chiefly to the involuntary functions, the ganglionic nerves being given to those organs over which the will has little or no power.

The remarks of Blumenbach on the VOLUNTARY MOTIONS are characterized by the same simplicity and acumen as we have seen throughout the parts of his work already noticed. He states the common division into voluntary and involuntary motions, but wisely observes:—

"If this division is narrowly examined, it will be found embarrassed by so many difficulties, that the limits of each class cannot be well determined. For, on the one hand, few functions can be termed truly involuntary, especially if we consider the connexion of the imagination and passions with the will. Again, on the other hand, there are few voluntary motions that may not be rendered involuntary by the force of habit, whose influence upon our animal motions is immense. Of the latter description are those muscular motions, which, although generally voluntary, take place, under certain circumstances, without the knowledge of the mind, or even in opposition to its endeavours. On the contrary, some muscles, which are almost always obedient to the will, cease, under some circumstances, to be so: an instance of this exists in the difficulty which we experience in attempting to move the hand and the foot of the *same* side *in different* directions, and in all those motions which, although voluntary and perfectly easy if produced separately, are found very difficult if attempted together."

Drs. Baynard and Cheyne saw an English officer, who could stop the action of his heart and arteries at pleasure. Rumination being possessed by some individuals, shews that the action of the stomach is sometimes voluntary.

These remarks and circumstances have been brought before the reader, in order that stickling for terms might not be made use of, as so frequently happens, as a barrier against the progress of truth. The more our knowledge extends, and the more we reflect on that knowledge, the more do we discover the necessity of not being positive in our opinions, and of not being attached too strongly to particular terms. Dr. Elliotson, however, has made an attempt to define voluntary and involuntary muscles.

"Those muscles, I conceive, are called voluntary, which we have ordinarily the power of directly contracting: those involuntary, which we have not ordinarily the power of directly contracting. These two definitions appear to me unexceptionable."

Part of the note A to this section, by Dr. Elliotson, is so interesting, that we shall present it to our readers.

"Prevost and Dumas assert, that the muscular fibres, straight while at rest, approximate each other at intervals, under contraction, so as to acquire a zigzag course ( \/\/\/ ), and shorten the distance of their two extreme points. They ascertained satisfactorily, that during contraction no increase of volume is acquired. If muscles, while the fibres are straight, are stretched still more, as continually happens in the muscular coats of cavities, the subsequent shrinking to the original dimensions, is unattended by the zigzag appearance. Nervous filaments, they also assert, go perpendicularly to the muscular fibre at the very points where the angles are formed under the zigzag contraction, and yet not to terminate there or unite with the muscular fibres, but to return or anastomose with other nerves. The approximation of the nervous filaments to each other is thought to draw the muscular fibres into angles, and thus be the cause of muscular contraction. The approximation of the nervous filaments is considered an electric phenomenon. Electricity will effect it, and in whatever way it is effected, electric appearances are said always to be discoverable.

"Muscular power is nowhere more displayed than in some fish. 'I have seen,' says Sir Gilbert Blane, 'the sword of a sword-fish sticking in a plank which it had penetrated from side to side; and when it is considered that the animal was then moving through a medium even a thousand times more dense than that through which a bird cleaves its course at different heights of the atmosphere, and that this was performed in the same direction with the ship, what a conception do we form of this display of muscular power!' Muscular strength is proportionably much greater in smaller animals. A flea can draw from seventy to eighty times its own weight, whereas a horse cannot draw with ease more than three times its own weight.'"

The next section is on Sleep, which Blumenbach considers to be dependent upon a diminution of the influx of blood to the brain. Dr. Elliotson differs from his friend, and thinks that this alteration in the circulation is not a cause, but a consequence. Taking this view, we can explain how sleep is dependent upon the weariness of the brain: it, like every other organ of the body, if, by stimulus, excited to increased action, must have a time of repose. "The case of the brain is analogous," says Dr. Elliotson, "and when, after its daily activity, it falls asleep, the diminution of its circulation consequently ensues. The influence of sleep upon the cerebral circulation is shown by the headache and other marks of congestion which follow too much sleep. Boerhaave mentions a physician, who took a fancy that sleep was the natural state of man, and so slept eighteen out of the twenty-four hours, till he died of apoplexy. The horizontal posture will not explain these ill effects, because persons with spinal disease will lie a year upon the back without them."

Dr. Elliotson then states some facts respecting the duration of sleep; explains somnambulism, and gives a short, but pleasing account of animal magnetism.

There is one circumstance connected with the last-mentioned subject, which is, that men of the greatest talent, Georget, Hufeland, Treviranus, Oken, Kieser, Carus, believe in it. This we have noticed, because it seems so much the vogue, in the present day, to approve or disapprove of doctrines, because Mr. Abernethy, or some other man of note, believes or disbelieves them. Great names can be brought forward in support of the most ridiculous hypotheses: indeed, there is no opinion, however foolish, but has had some philosopher to support it. Our favourite motto is "res non verba quæso." Indeed, we despise that submission of the understanding before the shrine of mere human opinion; the majesty of facts is the only one before which we can bow; we hold no man master—the ὁ εφη will no longer do.

Besides *animal* powers, the loss of which sleep repairs, we have *natural* powers, which are kept in existence by a due supply of substances placed in certain relations to our system as to nourish the same; substances taken *en masse* constituting what is called FOOD. This supply of food being, according to the constitution of our nature, absolutely necessary, the Creator has wisely implanted the feelings of HUNGER and THIRST, which impel us to seek the due supply.

Blumenbach, like every reasonable physiologist, maintains that man is *omnivorous*. Still, though man is omnivorous, he can live upon one thing only: thus, "the Nomadic Moors have scarcely any other food than gum senega; the inhabitants of Kamtschatka, and many other shores, scarcely any other than fish: the shepherds in the province of Caracas in South America, on the banks of the Oronoko, and even the Morlachs in Europe, live almost entirely on flesh." Some very striking facts relating to this subject are given in a note by the translator, 308, 309, 310, 311.

Having considered food generally, Blumenbach proceeds to consider those processes by which the food taken is rendered subservient to the uses of the animal machine. Of these, MASTICATION and DEGLUTITION are the two first mentioned. Of deglutition, Blumenbach gives a short but neat description, p. 313.

"The lower jaw is connected with the skull by a remarkable articulation, which holds a middle rank between arthrodia and ginglymus, and, being supplied with two cartilaginous menisci of considerable strength, has easy motion in every direction.

"The digaster, assisted somewhat by the geniohyoidei and mylohyoidei muscles, draws the lower jaw down, when we open the mouth.

"The masseters and temporal chiefly raise it again when we bite off any thing, and are most powerfully contracted when we break hard substances.

"Its lateral motions are accomplished by the internal and external pterygoid.

"The latter can also draw it forwards."

And at the next page, Blumenbach adds,—

"The mechanism of deglutition, although very complicated, and performed by the united powers of many very different parts, amounts to this. The tongue being drawn towards its root, swelling and growing rigid, receives the bolus of food upon its dorsum, which is drawn into a hollow form. The bolus is then rolled into the isthmus of the fauces, and caught with a curious and rather violent effort by the infundibulum of the pharynx, which is enlarged and in some measure drawn forward to receive it. The three constrictores muscles of the pharynx drive it into the œsophagus. These motions are all performed in very rapid succession, and require but a short space of time."

The next process is DIGESTION, the food having been received into the *stomach*. This organ is characterized very conspicuously by its being abundantly supplied with nerves, both from the *ganglionic* and from the *cerebral* system; also with blood-vessels. It seems, from the experiments of Dr. Philip, and the observations of Sir Everard Home, that the process of digestion goes on principally in the cardiac portion of the stomach; and, from the experiments of Dr. Prout, that muriatic acid is formed during the digestive process. Tiedemann and Gmelin maintain the same opinion.

Dr. Elliotson, in a note to this section, gives a short account of the stomachs of different animals; and also some new remarks on vomiting, to which we refer our readers.

Respecting the PANCREATIC JUICE, Blumenbach states, that "The excretion of this fluid is augmented by the same causes which affect that of saliva, pressure and stimulus;" and that "Its use is to dissolve the chyme, especially if imperfectly digested in the stomach, and at all times, by its great abundance, to assimilate the chyme more to the nature of the fluids, and render it fitter for chylification." Tiedemann and Gmelin, Dr. Elliotson states in a note, maintain that "this fluid animalizes the unazotised principles of vegetable food:" and adds, that "it is certainly much larger proportionately in herbivorous than in carnivorous animals."

There is a very curious note to this section, illustrative of the idea, that some animals have many lives.

"Brunner, about 150 years ago, removed almost the whole pancreas from dogs, and tied and cut away portions of the duct; and they lived apparently as well as before. From one he was not contented with removing the spleen at one time and the pancreas at another, after which the poor animal *pancratice valebat;* but, to render it celebrated for experiments, he on a third occasion laid bare the intestines and wounded them for an inch and a half, sewed up the wound, made a suture in the abdominal parietes so badly that the intestines were found hanging out on the ground one morning, purple and cold, and then allowed the animal to lick the wound into healing. He also performed the operation for aneurism in the artery of its hind leg, and paracentesis of its chest, injecting a quantity of milk into the pleura and pumping it out again. This even was not enough for the gentle Brunner; he gave the dog such a dose of opium, when it had recovered from the operation on the spleen, that it was seized with tetanus. But this also it got the better of, and lived upwards of three pleasant months with its master, 'gratus mihi fuit hospes,' after all these indulgencies, and was at last lost in a crowd; stolen, no doubt, because 'celebris ob experimentorum multitudinem,—vivum philosophiæ experimentalis exemplum, et splene mutilus, variis cicatricibus notabilis.' Brunner offered any money for it again, but to no purpose."

As a matter of great importance in the process of digestion, the BILE claims the consideration of every physiologist. The deficiency and the excess of this secretion, occasioning so many and so distressing symptoms, render a knowledge of the nature of the fluid itself, of the organ producing it, and of the powers influencing it, highly important. As to the opinion whether the bile be secreted from arterial or venous blood, Blumenbach remarks:—

"Although the former opinion is countenanced by the analogy of the other secretions which depend upon arterial blood, nevertheless more accurate investigation proves that the greater part, if not the whole, of the biliary secretion is venous.

"With respect to arguments derived from analogy, the vena portæ, resembling arteries in its distribution, may likewise bear a resemblance to them in function. Besides, the liver is analogous to the lungs, in which the great pulmonary vessels are intended for their function, and the bronchial arteries for their nourishment; and if we are not greatly mistaken, the use of the hepatic artery is similar. We would, however, by no means completely deny its importance in the secretion of bile, but must regard it as inconsiderable, adventitious, and not well established."

These observations are worthy of their source. How contrary to the childish observations of an individual, who, some how or other, has attained as a medical practitioner a fame, which seems to have no other origins than the efficacy of blue-pill and the eccentricity of his manner. This individual tells us with an air, as if giving birth to wisdom itself, as if a new Minerva was about to spring from his brains, "the function of the liver is a strange thing: it is what does not take place in any other part of the body." Surely this is the mountain labouring to bring forth a mouse. And then, with equal wisdom, brings forward another child of his own creation, and well worthy of its creator; namely, that bile is secreted from the arterial blood: and this doctrine, be it observed, the acute physiologist founds on *one* fact: and that fact, a *lusus naturæ!* Can it be believed that this man talks sometimes about Bacon, the author of the grand system of induction? A single fact to establish a doctrine! This is attempting "to build a ship with materials not sufficient to make a boat." No wonder that hypotheses should abound, when such a person sets such an example!

In a note to this section, Dr. Elliotson gives the results of the experiments of Dr. Blundel and others on the effects of tying the ductus communis choledochus. To the book itself we refer our readers, extracting, however, the following interesting portion:—

"The hypothesis, that one great use of the liver was, like that of the lungs, to remove carbon from the system, with this difference, that the alteration of the capacity of the air caused a reception of caloric into the blood, in the case of the lungs, while the hepatic excretion takes place without introduction of caloric,—was, I recollect, a great favourite with me when a student, principally from the facts that a supply of venous blood—blood which has been used by the system,—runs to both liver and lungs, and to no other organs; that the higher the temperature the less carbon passed off by the lungs (less caloric being demanded by the body), and the more abundant, or more acrid, became the bile; so that bilious diseases are most prevalent in hot seasons and climates. The Heidelberg Professors have adduced many arguments to the same effect. In the fœtus, for whose temperature the mother's heat must be sufficient, the lungs perform no function, but the liver is of great size, and bile is secreted abundantly, so that the meconium accumulates considerably during the latter months of pregnancy. We shall see, indeed, that at the very time the functions of the lungs suddenly begin at birth, the liver suddenly loses much of its supply of blood. Warm-blooded animals with large lungs, living in the air, have the liver proportionally smaller than those which live partly in water: in cold-blooded animals, and reptiles, which have lungs with such large cells as but slightly to decarbonise the blood; in fish, which get rid of carbon but slowly by the gills; and in the mollusca, which decarbonise still more slowly by gills or lungs,—the liver is proportionally large. More blood flows to the liver, accordingly as the lungs are less active organs. In the mammalia and birds it receives the blood of only the stomach, intestines, spleen, and pancreas; but in the cold-blooded, of many other parts; in the tortoise, of the hind legs, pelvis, tail, and vena azygos; in serpents, of the right renal, and all the intercostal veins; in fish, of the renal veins, the tail, and genitals. They assert, that in pneumonia and phthisis more bile is secreted, and in the *blue disease*, and other affections of the heart, that the liver is enlarged. The constituents of the bile contain a large quantity of carbon, which is chiefly in union with hydrogen, and under the form of resin or fatty matter, and resin is most abundant in the bile of herbivorous animals, whose food contains a very large proportion of carbon and hydrogen. In the lungs the carbon may be said to be burnt, whence animal heat; in the abdomen it passes off still combustible."

Blumenbach proceeds in the next section to the use of the SPLEEN; that which has had so many uses, but all for no use. He considers the two hypotheses as most worthy of notice to be these: that which considers the spleen as a diverticulum to the arterial blood destined to form the gastric juice; the other, that it affords a short passage to a great portion of our drink from the stomach into the mass of blood. This is all that a candid physiologist can at present say.

The uses of the OMENTUM are then taken notice of. Besides the commonly stated uses, lubricating the intestines and assisting their movements, Blumenbach states, "There is another two-fold office attributed with great probability to the omentum, viz. that of facilitating the dilation of the viscera, to which it is contiguous, and of acting as a diverticulum to their blood during their state of vacuity."

Blumenbach next describes the uses of the INTESTINES, after giving a short but clear account of their structure. He proves by a quotation that Fallopius was the first discoverer of the *valve* of the colon.—(*See note S. p.* 354.) One circumstance he notices which we shall mention. It is, that the large intestines, of which the peristaltic motion is weak, are so placed, as to experience the pressure of the abdominal parietes, this compensating for the other circumstance: a fact, shewing physiologically the importance of foot exercise.

In speaking of the ABSORBENT SYSTEM, which occupies the XXIX. Section, Blumenbach divides it into four parts, the *lacteal* and *lymphatic* vessels, *conglobate glands*, and the *thoracic duct*, each of which he describes succinctly. In the note (D) to this section, we have presented to us, by Dr. Elliotson, a brief view of the experiments on absorption. To this note we refer our readers, and also to the experiments of Dr. Barry on absorption, which, as matters of physiology, are highly instructive: and, as all truth is important, when our views on this point are more clear, our practice, perhaps, on some cases of disease of the absorbent system, may be less empirical.

The next subject is SANGUIFICATION, or the conversion or assimilation of the chyle to the blood, which is favoured by the windings in the mesenteric and other conglobate glands, and also by the slow, and as Blumenbach styles it, "the almost stillatitious manner in which the chyle joins the blood through the last valve of the thoracic duct, these very minute portions being thus the more intimately combined with the blood."

The chyle is often found in the other fluids of the body. Dr. Elliotson in a note says—

"I once saw a young married woman whose urine contained very large coagula of chyle. She always dined at noon. In the evening the coagula were white; in the morning pale with pink streaks. After fasting twenty-four hours at my request, the coagula still appeared in the urine, extremely pale, and showing more pink streaks. She had been some months in this way, was in very fair health, and had a great appetite, and perhaps some other general symptoms of diabetes; but there was no sugar in the urine. Notwithstanding the fluid discharged seemed to present as much coagulum as urine, the quantity of chyle proved on drying to be very minute, and from its looseness to have been extremely distended by the urine. As this was a state of disease, I draw no inference from the case respecting the time necessary for the change of chyle to blood. She would not allow me to take any blood from the arm for observation.

"I know that similar cases have been seen by Dr. Prout and other gentlemen now practising in London, and there may be several on record, but the only one of which I have read is quoted in Shenkius. 'I saw,' says the author whom he quotes, (in Castro Itri, Comitatus Sundorum,) 'a young man, thirty years of age, who daily made a considerable quantity of urine, depositing a white substance like the curd of milk, sufficient to fill a common *pot de chambre,* besides the urine which was above it. He was in perfect health, not experiencing the slightest ill effect.'

"Shenkius is generally thought a credulous collector of incredible cases, and no doubt some of his histories as well as of his opinions are ridiculous. But careful modern observation discovers facts precisely similar to the greater number that he has collected. I should have doubted the history just related, more especially the good health of the patient, had not the case of the woman occurred to me. He gives some instances of black urine made by persons in perfect health, and Dr. Marcet has published two such in the *Transactions of the Medical and Chirurgical Society*. Dr. Prout showed me a specimen of urine from one of these."

The next step is NUTRITION, which Blumenbach says "is the grandest gift of Nature, and the common and highest prerogative of the animal and vegetable kingdoms, by which they, beyond measure, surpass, even at first sight, all human machines and automatons."

Respecting the *reproductive power*, which forms again parts that have been destroyed, and which is continually effecting changes in the structure of the machine, Blumenbach, from his experiments on man and warm-blooded animals, has been led to this conclusion, that "it appears completely bestowed upon scarcely any similar solid part which *possesses any other vital power* BESIDES CONTRACTILITY, i. e. *irritability*, *sensibility*, or *a vita propria.*

The power of reproduction is much more considerable in animals than in man. Our physiologist observed the reproduction of the whole head with its four horns in a snail, and the complete eye—cornea, iris, crystalline lens, &c. in a water-newt. Besides reproduction, reparation is also proportionably great. Brunner's dog testifies to this.

---

We now have arrived at the third division of the fluids of the body by this celebrated physiologist; namely, the SECRETED, which he ushers in by some general remarks on the SECRETIONS. Of the fluids themselves he gives an arrangement founded on the degree of difference between them and the blood from which they are formed. The following is his order: *milk*, *aqueous fluids*, such as tears, vapour in the cellular interstices, cavities of the abdomen and thorax, the fluid of the pericardium and of the ventricles of the brain; the liquor amnii, and the urine. Next come the *salivary fluids;* next, the *mucous*, lining the cavities of the organs performing the natural and genital functions, and likewise the tract of the nostrils, larynx, and trachea: the *adipose*, as the common fat, medulla of the bones, grease of the skin, the secretion of the corona glandis, and of the external female genitals; and, finally, the truly *serous* or albuminous, as the fluid of the ovarian vesicles of De Graaf, and the liquor of the prostate. "The *semen virile* and the *bile* are each *sui generis.*"

The modes of secretion are then stated; the most simple being diapedesis or transudation; the next more complicated, secretion by *glands*. The following passage is worthy of notice:—

"Properly speaking, the *conglomerate* (as they are called to distinguish them from the lymphatic conglobate) are the only true secreting organs: such as the salivary and lachrymal glands, the pancreas and breasts. They are provided with an excretory duct coming immediately from the large lobes, which are composed of others, smaller, and whose interior structure was once the source of warm disputes in the schools of medicine. Malpighi considered the miliary globules, which are easily discoverable in most glands, as acini, according to his expression, internally excavated. Ruysch, on the contrary, contended that these supposed hollow acini were nothing more than glomerules of blood-vessels,—an opinion shown to be far more consistent with nature by microscopical observation and the effects of minute injection.

"The structure of some secreting organs, especially of the liver and kidneys, the latter of which strikingly exhibit the glomerules of Ruysch or the acini of Malpighi, are not, excepting in their peculiar parenchyma, very dissimilar from this structure, and indeed throw considerable light upon the question. On the outer part of these, small twigs arise from the sides of the capillary arteries and run into vascular glomerules, hanging from them like granules as from stalks: from these arterial glomerules spring both very minute colourless secreting vessels whose origin from the extremities of arteries were formerly alluded to (92), and the radicles of veins into which the arteries are continued, and which convey back into the venous trunks the remaining blood deprived of the secreted fluid."

The organization of the testes is peculiar to itself.

As to the absolute cause of most secretions, Blumenbach rightly refers it to the intimate structure of the secreting organs, and also to the parenchyma, which, he thinks, is possessed of a *vita propria*, "a peculiar species of vitality distinct from the common vital powers of contractility, irritability, and sensibility."

Fat is next considered. This, in the form of drops, is deposited between the lamina of the mucous tela. The two theories respecting the formation of fat are mentioned: namely, that it is formed by peculiar glands, and that it merely transudes from the arteries. Blumenbach inclines to the latter opinion, because fat sometimes exists as a diseased formation in parts naturally destitute of it.

The Excrementitious fluids, or the excrements of digestion, as Blumenbach calls them, are of two kinds, the one exhaled by perspiration; the other, the urine. Having described the kidneys and the bladder, Blumenbach states the way by which the urine is expelled; "The bladder is evacuated from the contraction of the sphincter being overcome by the action of the detrusor urinæ and by the pressure of the abdomen. To these in men is superadded the action of the accelerators, which force out even the drops of urine remaining in the bulb of the urethra."

We now take leave of the fluids of the body, and are introduced, in the XXXV. Section, to the General Differences of the Sexes. These are briefly stated; those peculiarly claiming the attention being the *particular* differences, namely, the genital organs, being in man fitted for impregnating, in woman for conceiving. This section is enriched by Dr. Elliotson's notes: first, upon Sir Everard Home's hypothesis, that the sex is not determined at the first formation of the individual; next, upon the difference of form, mental powers, &c. of men and women; and, finally, on hermaphrodites and malformations in general. In these notes there is abundant and curious information; they testify to the research of the writer, and show, by the arrangement of the facts, his powers of so ordering his thoughts, that one is necessarily connected with the other.

Leaving these notes to be perused in the work itself, we proceed to the consideration of the next section; namely, THE GENITAL FUNCTION IN MAN. The observations of Blumenbach upon the situation and descent of the testicles of the fœtus are so acute, somewhat novel, and so clear, that they may justly be quoted.

"On opening the lower part of the abdomen of a young fœtus, there appears in each groin, at the ring of the oblique muscles, a very small opening in the peritonæum, leading downwards to a *narrow passage* which perforates the ring, and runs to a peculiar sac that is extended beyond the abdominal cavity towards the scrotum, is interwoven with cellular fibres, and destined for the future reception of the testicle.

"At the posterior margin of this abdominal opening, there is sent off another process of peritonæum, running upwards, and appearing, in the young fœtus, little more than a longitudinal fold, from the base of which arises a small cylinder, or rather an inverted cone, that terminates above in a globular sac, containing the testis and epididymis, so that the testis, at first sight, resembles a small berry resting on its stalk, and appears hanging, like the liver or spleen, into the abdomen.

"The vessels, which afterwards constitute the spermatic chord, are seen running behind the very delicate and pellucid peritonæum; the spermatic artery and vein descending along the sides of the spine, and the vas deferens passing inwards, in the loose cellular substance behind the peritonæum, towards the neck of the bladder. They enter the testis in the fold of peritonæum just mentioned.

"After about the middle period of pregnancy, the testis gradually descends and approaches the narrow passage before spoken of (511), the fold of peritonæum and its cylinder becoming at the same time bent down, until it lies directly over the opening of the passage.

"The testis being now ready for descent, the opening which was hitherto small, becomes dilated, so as to allow the organ to pass it, the abdominal ring, and the whole passage, and to descend into the bulbous sac (511); after this occurrence, the opening soon becomes strongly closed and even grows together, leaving scarcely any vestige of itself in infancy.

"In proportion to the slowness with which the testis proceeded towards the opening, does its transit through the abdominal passage appear rapid, and, as it were, instantaneous. It is common to find the testis in mature fœtuses either lying over the peritonæal opening, or, having passed this, resting in the groin; but I have once only met with the testis, and then it happened to be the right and in a twin fœtus, at the very time when it was adhering, and in a manner strangled, in the middle of the passage, being just about to enter the sac; in this instance, the left testis had passed the abdominal canal and was already in the sac, and the abdominal opening of the same side was perfectly closed.

"This remarkable passage of the testis from the abdomen through the groin is limited to no period, but would seem to occur generally about the last month of pregnancy; the testicles are found, however, not very rarely in the abdomen or the upper portion of the groin at birth. For they have always another part of their course to finish, after leaving the abdomen, viz. to descend, together with their sac, from the groin into the scrotum."

This we present to our readers as a master-piece in description: as an example of correct writing, which medical authors would do well to imitate. It is one of those passages which will bear Addison's test of a good style, namely, that no addition, no subtraction can be made to or from it.

Blumenbach doubts whether genuine semen is ever absorbed during health. Nocturnal pollutions, as they are improperly called, our physiologist considers as a natural excretion intended to liberate the system from the otherwise urgent superfluous semen. The laconic brevity of the following passage is great.

"The emission of semen is *excited* by its abundance in the vesicles and by sexual instinct; it is *effected* by the violent tentigo which obstructs the course of the urine, and, as it were, throws the way open for the semen; by a kind of spasmodic contraction of the vesiculæ seminales; by a convulsion of the levatores ani, and of the acceleratores urinæ; and by a succussion of the whole system, short and less violent, though almost of an epileptic nature and followed by depression of strength."

Some excellent notes are affixed to this section; our limits oblige us to refrain from quoting them.

The GENITAL FUNCTION OF WOMAN is next brought forward. The description of the parts concerned is brief and clear, and those referring to the muscularity of the uterus we shall quote.

"I have never yet discovered a true muscular fibre in any human uterus which I have dissected, whether impregnated or unimpregnated, recent or prepared; but it must be allowed by those who maintain the muscularity of the uterus, that the fibres, which they call muscular, have qualities very different from those of all others in the system, especially since they themselves entertain doubts of the existence of nerves in the substance of the uterus, without which, one cannot imagine a true muscle. (302) I am daily more convinced, that the uterus has no true irritability, (301) but, if any part of the body has, a vita propria, (42) perfectly correspondent with the peculiar motions and functions of the uterus, which are not referable to any properties common to the similar parts, (39-41) and which appeared to the ancient physicians and philosophers so peculiar, that the uterus was by them denominated, an animal within an animal."

Blumenbach refers to female circumcision, and Dr. Elliotson gives a note on this subject, which may be introduced to the reader's notice, not with the view of imitation, but simply to show the powers of nature, and the punishments to which jealousy subjects individuals.

"This custom is mentioned even by Strabo. (p. 284.) Burckhardt states that, 'the daughters of the Arabs, Ababde and Djaafere, who are of Arabian origin, and inhabit the western bank of the Nile, from Thebes, as high as the cataracts, and generally those of all the people to the south of Kenne and Esne, as far as Sennaar, undergo circumcision, or rather excision of the clitoris, at the age of from three to six years.' The healing of the wound is contrived to close the genitals, excepting at one point for the passage of the urine, and as the adhesions are not broken through till the day before marriage, and then in the presence and with the assistance of the intended bridegroom himself, no doubts of the fair's virginity can harass his breast.

"The same traveller, as well as Browne and Frank, relates that many slave girls have their genitals sewn up, and, like eunuchs, become more valuable on account of their unfitness for sexual connexion. 'Mihi contigit,' says he, 'nigram quandam puellam, quæ hanc operationem subierat, inspicere. Labia pudendi acu et filo consuta mihi plane detecta fuere, foramine angusto in meatum urinæ relicto.' He adds, 'Apud Esne, Siout, et Cairo, tonsores sunt, qui obstructionem novacula amovent, sed vulnus haud raro lethale evenit.'"

From a note given by Dr. Elliotson, we find that Mr. C. Bell maintains the muscularity of the uterus: and his views upon this point are quoted from the Med. Chir. Trans.

Our physiologist devotes his 38th section to the MENSTRUA, a fluid poured out from the uterus, monthly, during about 30 years; "a law," as he says, "imposed upon no other species of animal." "The proximate cause is supposed to be a *local* plethoric congestion." The concluding remarks are well timed. "It will be better to confess our ignorance of the cause of its periodical return, than to indulge in vain hypotheses; for all the periodical phenomena of health and disease, *that continue more than twenty-four hours*, have hitherto appeared among the mysteries of animal nature."

In the notes to this section, some very interesting facts are stated with regard to menstruation; especially those related by Dr. Gall, which are deserving the attention of every practitioner.

It was the ordination of things of old, by the Creator, that from pairs of individual species, the multiplication of the species should take place. In fulfilling this, his will, he implanted the sexual desire, which excites the male and female to bring themselves into such relations one to the other, that the effect shall be a new being. With respect to the effect of the part, called the *placenta*, in nourishing this new being, Blumenbach has the following observations.

"After all, the truth appears to be this,—that no anastomosis exists between the blood-vessels of the uterus and of the chord, but that the oxygenised blood which proceeds from the uterus to the portion of the placenta that was originally the decidua crassa, is absorbed by the extreme radicles of the umbilical vein distributed upon the mossy chorion, and carried to the great venous trunk of the chord; while the carbonised blood returning from the fœtus, through the umbilical arteries, being poured in the same manner into the substance of the placenta, is absorbed by the venous radicles of the uterine portion of the placenta, and returned to the uterus."

The changes in the uterus after impregnation, during the ten lunar months, are very clearly stated. But to this we cannot do justice, unless we were to quote the whole. The same may be said of the notes, which abound with the most valuable matter, containing the testimonies of different physiologists to the increased action, induced by the excessive redness of the genital organs of the female, during heat. We are, in addition, presented with facts, shewing that impregnation may take place without the hymen being ruptured; with the experiments of Dr. Haighton, Dr. Blundel, and others.

There is one idea that we cannot pass by without notice: this is, that different conceptions may occur from the repetition of copulation after very short intervals. This is said to be proved by adulterous women, who have brought forth twins resembling different fathers in the colour of their skin; namely, black women who have brought forth a black and a mulatto; and European women, who have brought forth a white and a mulatto. Dr. Elliotson gives in note (B) some facts proving the truth of this idea.

The connexion between the uterus and breasts, seems to depend upon the anastomotic connexion between the epigastric and internal mammary artery. This anastomosis, existing in man as well as in women, we might infer, that milk may be afforded, even by males, and Dr. Elliotson gives us some examples in note (C) p. 508—509. Many similar cases with respect to animals are related. An opportunity is afforded in so doing, of sprinkling a little classic salt. "Blumenbach has described a he goat, which it was necessary to milk every other day, for a year; so that, to say with Virgil, *mulgeat hircos* is not tantamount to calling a man a fool."

Our physiologist, in the next section, rightly observes:—"From what has been said relatively to the functions of the fœtus, still contained within its mother, and immersed, as it were, in a warm bath, there must evidently be a considerable difference between its functions, and those of the child that is born, and capable of exerting its will." The differences are briefly narrated. The great size of the glands is a very conspicuous point of difference between the fœtus and the child after birth; and perhaps the investigation of the circumstances therewith connected might lead to some very important conclusions.

This valuable work is concluded by a section on the GROWTH, STATIONARY

CONDITION, and DECREASE of this being, which, by the processes before-mentioned, has been created. Blumenbach gives a neat description of the gradual and progressive steps through which the human embryo passes: he considers the *commencement of formation* to take place about the third week from conception; the *formation of bone* at the seventh or eighth week. The changes go on till death comes: *death without disease;* the physiological death, the *ευθανασια*. A curious circumstance is noted by our author, namely, that in the dissection of moribund mammalia, "the struggle of the heart may be perceived, and the right ventricle and auricle are found to live rather longer than the left."

The limit of the natural period of life, Blumenbach acknowledges it is scarcely possible to state, but considers, that a large proportion of Europeans reach their *eighty-fourth* year, and but few exceed this period.

We shall quote our physiologist's concluding period, as we hope it may have the effect of stopping the palsied complaints of some concerning the shortness of human life.

"But, on the whole, notwithstanding the weakness of children, the intemperance of adults, the violence of diseases, the fatality of accidents, and many other circumstances, prevent more than about perhaps seventy-eight persons out of a thousand from dying of old age, without disease; nevertheless, if *human longevity* be compared, *cæteris paribus,* with the duration of the life of any other known animal among the mammalia, we shall find that, of all the unreasonable complaints about the misery of human life, no one is more unfounded than that which we commonly hear respecting the shortness of its duration."

We have thus come to the conclusion of this invaluable work, so far as regards Blumenbach's share in it. We have seen throughout the philosophical physiologist; the modest writer, and the well informed man. We discover a candour, the product of enlightened honesty, throughout: a grasp of mind, that can take in the various particulars necessary to form a proposition: and an acuteness of intellect clever in detecting what pertains to the object in view, from that which does not. We perceive a careful and wise neglect of the egotistical *I*: the subject is the thing to be promoted, not the individual. The lover of truth is associated with the truth he loves: and goes down to posterity, when thus associated, in the best company. When thus linked, indeed, he may say, "exegi monumentum perennius ære." Such will be the case with Blumenbach.

We shall conclude our remarks by recommending this work to every individual in the medical profession. To the practitioner we would recommend it—because he has much knowledge presented to him in a small compass, and in a style, not adorned with the ephemeral flowers of a school boy's flimsiness, but grand in the dignity of its majestic solidity and worth; a style, that will suit the man who husbands his time, and who *thinks;* who delights in food, that will afford some nutriment. Besides, there is a spirit gone out into the world; a spirit, which is raising men, and pushing them on in the career of intellect; a spirit which will make its possessor take the practitioner's place, unless the practitioner keeps pace with those now close upon his heels. Mystery will do no longer: practice *must* be founded on science; physiology *must* be understood; and medical practitioners *must* be ready to give a reason for their practice.

To the medical writer we recommend this work as a true specimen of the style in which medical works should be written. We are made angry at perceiving the flimsy productions often presented to our notice; indeed, with many writers, the only aim seems to be to expand into twenty lines, a thought, which is compressible into twenty words; and thus the time of the reader is sacrificed; the reader seeks a substance, and embraces a shadow. Let medical writers study Blumenbach; let them acquire the terse brevity of his style, the manly dignity of his expressions, and then they shall have our praise, and they shall be remembered by posterity. One parting remark.—It would be well before they begin to write at all, if they would scan over the notes of Blumenbach, and see how many works he has perused, how much knowledge he must have accumulated, before he wrote.

Finally, to the medical student we present this work with joy. When students ourselves, we adopted the following plan, of the uses of which we are now fully convinced. We read few works, but these were standards. We found from some good and clever medical friend what works are truly useful; what might, as Lord Bacon says, "be chewed and digested." Having found these, we chewed and digested them, and now find that we have been nourished. One work on one subject well studied will do more for an individual than reading twenty volumes. Reading does not make a work read the reader's property. It is *thinking*. On physiology, then, as a work capable of affording true nutriment to the medical student, we recommend Blumenbach's, translated by Dr. Elliotson. Let the student interleave his volume and add his own illustrations; put down his own thoughts; state his own difficulties: and then, after doing this, he will thank us for our advice; will acknowledge us as friends to him, friends to the profession, and friends to human society in general.

We now bid farewell to Blumenbach as *reviewers*, but not as *readers*; and, in bidding farewell, state our hope that, ere long, this work will be seen in the library of every medical student.

---

Before closing this review it seems necessary that some observations should be made upon the additional matter at the end of the volume given us by Dr. Elliotson. The reader has already been introduced to this individual, and will not tire, we are sure, in having some of Dr. E's. mental products presented to his mind.

To the last section of Blumenbach Dr. E. attaches some notes. He makes some remarks on the changes of the system induced at the period of puberty, and shews, by some facts, how dependent these changes are upon the development of the testes in the male and of the ovaria in the female. The influence is well known as regarding brutes: as every one is aware of the differences induced in them by castration. On the human species the changes are equally clear. In Egypt it is the custom to emasculate boys, whose faces, Burckhardt states, are "almost destitute of flesh, the eyes hollow, the cheek-bones prominent, and the whole physiognomy has a skeleton-like appearance." The operation there is usually performed between the eighth and twelfth year. Further illustrations are given at p. 530.

With respect to the dependence of the *number* of fœtuses upon the ovaria, the experiment of John Hunter is quoted.

"He took two young sows, in all respects similar to each other, and, after removing an ovarium from one, admitted a boar of the same farrow to each, and allowed them to breed. The perfect sow bred till she was about eight years old,—a period of almost six years, in which time she had thirteen farrows, and in all one hundred and sixty-two pigs; the other bred till she was six years old,—during a space of more than four years, and in that time she had eight farrows and in all seventy-six pigs. Thus it would appear that each ovarium is destined to afford a certain number only of fœtuses, and that the removal of one, although it does not influence the number of fœtuses produced by the other, causes them to be produced in a shorter time."

Dr. Elliotson then proceeds to the examination of the opinion, very generally received, that the sexual desire depends upon the sexual organs. He disputes this, and advances an opposite opinion, that this desire is dependent upon the cerebellum; this latter view, from some diligent consideration, we are inclined to adopt, and our reasons cannot be better expressed than in the words of this distinguished physician.

"The sexual organs are usually regarded as the cause of sexual desire. That this is not the case, may be shown by many circumstances. Desire is by no means commensurate with the size of the genitals: even when the genitals are precociously developed desire is sometimes not felt. Desire is often felt after the removal of the testes, and in old age when the genitals are powerless. It must, therefore, depend upon some other part. This part appears to be the cerebellum. Desire is, *cæteris paribus*, naturally strong or weak in the adult, in proportion to the large or small size of the cerebellum, whether of the lobes or the fundamental portion called the vermiform process, which alone exists in birds, amphibia, fish, and insects; whenever I have accurately known the strength of the sexual propensities in either sex, the size of the occiput has without a single exception corresponded. Before puberty the cerebellum is small; its proportion in size to the cerebrum is at birth from one-ninth to one-twentieth, or even less: in the adult it is as one fifth, or at the least as one-seventh, and acquires its full development between the eighteenth and twenty-sixth years; and the breadth and prominence of the occiput are proportional. In old age, the cerebellum shrinks, and the internal table of the occipital bone following, bony matter is deposited between the two tables, and the bone at the fossæ occipitales becomes much less transparent. Gall possesses old crania in which the cerebellum had returned to the dimensions of infancy, and the occipital fossæ had become shallow. When the cerebellum is precociously developed, desire is felt by the child, even though the genitals are not above the ordinary size. Inflammation and irritation of the cerebellum are found by a multitude of dissections to have existed when great excitement of the genitals occurred before death, and injuries of the cerebellum, at the back of the head, have as frequently occasioned impotence. Desire is much stronger in the males of all species than in the females; and, in general, the cerebellum of the male is larger than of the female,—the distance between the mastoid processes is wider; the back of the neck and head, fuller; indeed the whole is much thicker; and if the brains of the two sexes are placed in water, the larger cerebellum of the male is very conspicuous.

"The sympathy of the cerebellum with the genitals, is the reason of the latter being regarded as the seat of desire. If they are removed, desire is generally extinguished; for the cerebellum is not afterwards developed at puberty, and the back of the head and neck remains small, perhaps smaller than in the female. If one testicle only is removed, Gall has invariably observed, in experiments on rabbits, and some cases in the human subject, that the opposite half of the cerebellum is not developed or shrinks. Removal of both or one testicle after puberty produces sometimes exactly similar effects. On the other hand, morbid irritation of the genitals will sometimes excite intense desire; and, judging from all the other facts, we should say from exciting the cerebellum. In violent sexual excitement, the back of the neck is flushed, and hotter. Some animals feel the sexual desire at certain periods of the year only; and at this time the testes, and in some instances the vesiculæ seminales and prostate gland, enlarge very considerably, as in the male sparrow and frog. Gall found the cerebellum of birds collected at this season, broader and more turgid, and the corresponding prominences of the cranium manifestly greater than in those collected at the beginning of winter.

"The facts adduced by Gall, on these points, in the third volume of his octavo work, are curious and very numerous; and similar ones, without end, may be found in works upon disease, military surgery, and physiology, from ancient times down to Magendie's Journal for January last year. It occasionally happens, that apoplexy or other disease of the cerebellum, is not attended by affections of the genitals; and

I am inclined to believe, that when no excitement of those organs accompanies the disease of the cerebellum, the disease does not include the vermiform process, which is considered by Gall the fundamental part of the cerebellum, from its being the only part always existing in animals where there is a cerebellum."

Speaking of the longevity of some individuals, Dr. E. states a circumstance regarding Parr, who retained his vigour to a hundred and forty, and died at a hundred and fifty-two, from plethora, induced by a change in his diet.

"At 105, he did penance in a white sheet for an illicit amour, which physiological fact John Taylor the poet, in 1635, immortalized in the following elegant rhymes:—

"Fair Catharine Milton was this beauty bright,
Fair like an angel, but in weight too light,
Whose fervent feature did inflame so far
The ardent fervor of old Thomas Parr,
That for love's satisfaction 'twas thought meet
He should be purged by standing in a sheet;
Which aged he *one hundred and five* year,
In Aldersbury Bury's church did wear.
Should all that so offend such penance do,
Oh! what a price would linen rise unto,
All would be turn'd to sheets, our shirt and s———k,
Our table linen, very porter's frock,
Would hardly 'scape transforming. *Eccentric Mirror*, vol. i."

Thus we are brought to a conclusion of the notes to this work.

---

It is almost impossible, at times, to trace the series of associated ideas which give rise to a new and a beautiful thought. Still sometimes it is possible, and when we can gradually go back, step by step, to the source of our original idea, we feel much delighted. Cannot the reader imagine it to be very natural that, after thus going over the physiology of MAN, the thought, the sublime thought, of viewing man but as a link in the great chain of creation, would arise in the mind. We think so; and this so happened with Dr. Elliotson: for, as the summing up of all this excellent matter, he gives us what we shall express in his own words. "The functions of the human machine having now been fully described, it may be useful to consider it in relation to other systems, and to review the chief varieties in which it appears."

This is an apologue to a very pleasing essay, in which the essayist first ably shews, that the commonly-received notion, about the regular gradation of beings from the great first cause down to inanimate matter, is not true: in other words, that the gradation is not regular, not insensible.

Dr. Elliotson maintains a truth which Nature maintains, and, consequently, what every true philosopher must maintain, *that animals enjoy* MIND. The possession of mind, Dr. E. asserts, necessarily implies the presence of a brain for its exertion, and introduces a note quoted from Blumenbach's Comparative Anatomy, wherein it is stated by that celebrated physiologist, that those animals which inhabit corals, and the proper zoophytes, most genera of the other orders of the Linnæan class of vermes, are found to possess a distinct nervous system, although, as he adds, "former anatomists have expressly declared, in several instances, that no such parts existed."

Dr. Elliotson gives a very striking instance of what might be called judgment, in a wasp, which would be so named if exhibited by a man. It is quoted from Dr. Darwin's Zoonomia.

"'A wasp on a gravel walk had caught a fly nearly as large as itself. Kneeling on the ground, I observed him separate the tail and the head from the body part to which the wings were attached. He then took the body part in his paws and rose about two feet from the ground with it; but a gentle breeze wafting the wings of the fly turned him round in the air and he settled again with his prey upon the gravel. I then distinctly observed him cut off with his mouth first one of the wings, and then the other, after which he flew away with it, unmolested by the wind.'"

The characteristics of mankind are well described. It is a subject admitting of an elevation of style, and Dr. E. does justice to it. His remarks on the erect posture we shall quote, as there is much useful information contained therein.

"The erect posture is natural and peculiar to man. All nations walk erect, and, among those individuals who have been discovered in a wild and solitary state, there is no well authenticated instance of one whose progression was on all-fours. If we attempt this mode of progression, we move either on the knees or the points of the toes, throwing the legs obliquely back to a considerable distance; we find ourselves insecure and uneasy; our eyes instead of looking forwards are directed to the ground; and the openings of the nostrils are no longer at the lower part of the nose,—in a situation to receive ascending odorous particles, but lie behind it. Our inferior extremities, being of much greater length, in proportion to the others and to the trunk, than the posterior of brutes with four extremities, even in children in whom the proportion is less, are evidently not intended to coincide with them in movement; they are much stronger than the arms, obviously for the purpose of great support: the presence of calves, which are found in man alone, shows that the legs are to support and move the whole machine; the thigh bones are in the same line with the trunk, in quadrupeds they form an angle, frequently an acute one; the bones of the tarsus become hard and perfect sooner than those of the carpus, because strengh of leg is required for standing and walking sooner than strength of arm and hand for labour; the great toe is of the highest importance to the erect posture, and bestowed exclusively on mankind; the os calcis is very large, particularly at its posterior projection, for the insertion of the strong muscles of the calf, and lies at right angles with the leg; we alone can rest fully upon it, and in fact upon the whole of the tarsus, metatarsus, and toes. The superior extremities do not lie under the trunk as they would if destined for its support, but on its sides, capable of motion in every direction towards objects; the fore-arm extends itself outwards, not forwards, as in quadrupeds, where it is an organ of progression; the hand is fixed not at right angles with the arm, as an instrument of support, but in the same line, and cannot be extended to a right angle without painfully stretching the flexor tendons; the superior extremity is calculated in the erect posture for seizing and handling objects, by the freedom of its motions, by the great length of the fingers above that of the toes, and by the existence of the thumb, which, standing at a distance from the fingers and bending towards them, acts as an opponent, while the great toe is, like the rest, too short for apprehension, stands in the same line with them, and moves in the same direction: were our hands employed in the horizontal posture, they would be lost to us as grand instruments in the exercise of our mental superiority. Quadrupeds have a strong ligament at the back of the neck to sustain the head; in us there is no such thing, and our extensor muscles at the back of the neck are comparatively very weak. They have the thorax deep and narrow, that the anterior extremities may lie near together and give more support; the sternum too is longer, and the ribs extend considerably towards the pelvis to maintain the incumbent viscera; our thorax is broad from side to side, that the arms being thrown to a distance may have greater extent of motion, and shallow from the sternum to the spine; and the abdominal viscera, pressing towards the pelvis rather than towards the surface of the abdomen in the erect attitude, do not here require an osseous support. The pelvis is beautifully adapted in us for supporting the bowels in the erect posture; it is extremely expanded, and the sacrum and os coccygis bend forwards below: in brutes it does not merit the name of pelvis; for, not having to support the abdominal contents, it is narrow, and the sacrum inclines but little to the pubes. The nates, besides extending the pelvis upon the thigh bones in the erect state of standing or walking, allow us to rest while awake in the sitting posture, in which, the head and trunk being still erect, our organs of sense have their proper direction equally as in walking or standing: were we compelled to lie down like quadrupeds, when resting during the waking state, the different organs of the face must change their present situation to retain their present utility, no less than if we were compelled to adopt the horizontal progression; and, conversely, were their situation so changed, the provision for the sitting posture would be comparatively useless."

Dr. E. then considers the erroneousness of different opinions regarding the brain as indicative of the mind; first, that which taught that the brain of man was the largest of all brains: next that of Söemmering, that the brain, in respect to the nerves arising from it, is the largest; that of Camper's facial line; that of Daubenton's occipital angle. All these are quickly disposed of, and for an account of their disposal we must refer the reader to the work itself.

Dr. E. then treats of the varieties of mankind adopting the division of Blumenbach already noticed. The Caucasian is thought to be pre-eminent in all the mental and corporeal particulars which distinguish man from brutes. He afterwards inquires whether these varieties have originated from the influence of various causes upon the descendants of two or of more parents, or to original differences in more than two primary parents? The replies to these questions must be taken in full; and, therefore, we should do injustice to the essayist to make quotations. We are tempted, however, to introduce one.

"*Analogical and direct facts* lead to the conclusion that none of the differences among mankind are so great as to require the belief of their originality.

"Animated beings have a general tendency to produce offspring resembling themselves, in both mental and corporeal qualities.

'Fortes creantur fortibus et bonis;
Est in juvencis, est in equis patrum
Virtus: nec imbellem feroces
Progenerant aquilæ columbam.'

"An exception occasionally occurs, much more frequently, we are told, in the domestic than the wild state,—the offspring differs in some particular from the parents; and by the force of the general tendency transmits to its offspring its own peculiarity. By selecting such examples, a breed peculiar in colour, figure, the form of some one part, or in some mental quality, may be produced. Thus, by killing all the black individuals which appear among our sheep, and breeding from the white only, our flocks are white; while, by an opposite practice pursued in some countries, they are black: thus a ram accidentally produced on a farm in Connecticut, with elbow-shaped fore-legs and a great shortness and weakness of joint indeed in all four extremities, was selected for propagation, and the ἀγκὼν breed, unable to climb over fences, is now established: thus some breeds of hares have horns like the roebuck: the Dorking fowl has two hind claws; and fowls in short are bred in every conceivable variety. Individuals, distinguished from others by no greater differences than those which thus spring up accidentally, cannot be supposed to belong to a separate species. Upon the comparison of these differences depends the analogical argument first employed by Blumenbach. Finding the ferret (*mustela furo*) to differ from the pole-cat (*m. putorius*) by the redness of its eyes, he concludes it is merely a variety of the same species, because instances of this deviation are known to occur accidentally in other animals; but he concludes the African elephant is of a species distinct from the Asiatic, because the invariable difference of their molar teeth is of a description which naturalists have never found accidental. Now there exist among mankind no differences greater than what happen occasionally in separate species of brutes."

Taking the whole work, we are glad to see two such clever men helping one another: one throwing light upon the views of the other: both good, though their goodness consists in points considerably different. The one is short and general; the other is fonder of detail and more full. Both are mutually helpful; and we leave the work, which has afforded us so much pleasure, with the hope that it may be soon undergoing another edition.
